# A Nanobody-Based Immunoassay for Detection of Ustilaginoidins in Rice Samples

**DOI:** 10.3390/toxins14100659

**Published:** 2022-09-23

**Authors:** Weixuan Wang, Gan Gu, Ruya Yin, Jiajin Fu, Mingpeng Jing, Zhen Shen, Daowan Lai, Baomin Wang, Ligang Zhou

**Affiliations:** 1 State Key Laboratory of Agrobiotechnology, Department of Plant Pathology, College of Plant Protection, China Agricultural University, Beijing 100193, China; 2 College of Agronomy and Biotechnology, China Agricultural University, Beijing 100193, China

**Keywords:** rice false smut, *Villosiclava virens*, ustilaginoidins, mycotoxins, variable domain of the heavy chain of heavy-chain antibody, nanobody, modeling, docking

## Abstract

Ustilaginoidins are a class of bis-naphtho-γ-pyrone mycotoxins produced by the pathogen *Villosiclava virens* of rice false smut, which has recently become one of the most devastating diseases in rice-growing regions worldwide. In this research, the nanobody phage display library was established after an alpaca was immunized with the hemiustilaginoidin F-hapten coupled with bovine serum albumin (BSA). Heterologous antigen selection and combing trypsin with competition alternant elution methods were performed for nanobody screening. Two nanobodies, namely, Nb-B15 and Nb–C21, were selected for the establishment of indirect competitive enzyme-linked immunosorbent assays (ic-ELISAs). For Nb–B15 and Nb-C21, their IC_50_ values were 11.86 μg/mL and 11.22 μg/mL, and the detection ranges were at 3.41–19.98 μg/mL and 1.17–32.13 μg/mL, respectively. Two nanobodies had a broad spectrum to quantify the contents of total ustilaginoidins in rice samples according to cross-reactivity. The recognition mechanisms of Nb-B15 and Nb-C21 against ustilaginoidin A were elucidated by molecular modeling and docking. The key amino acid sites for the binding of Nb–B15 or Nb–C21 to ustilaginoidin A were mainly located in the FR1 and CDR1 regions. As Nb-B15 was superior to Nb–C21 in the aspects of protein expression, ELISA titer, and tolerance to organic solvents, it was selected for application in the detection of actual contaminated rice samples. The total ustilaginoidin contents of rice samples were analyzed by Nb–B15-based ic–ELISA and HPLC-DAD, between which the results were found to be consistent. The developed immunoassay based on the nanobody from the alpaca can be employed as a rapid and effective method for detection of total utilaginoidins in contaminated rice samples.

## 1. Introduction

Mycotoxins are toxic secondary metabolites mainly generated by the fungal species of *Aspergillus*, *Penicillium*, and *Fusarium* [1,2]. Aflatoxins, ochratoxin A, fumonisins, T-2/HT-2 toxins, deoxynivalenol, zearalenone, citrinin, patulin, and ergot alkaloids are very common mycotoxins that have severe health risks to humans and animals. These mycotoxins are usually regulated by most countries and authorities [3,4,5]. Ustilaginoidins are bis-naphtho-γ-pyrone mycotoxins produced by *Villosiclava virens* (anamorph: *Ustilaginoidea virens*), the fungal pathogen of rice false smut (RFS), one of the grain-destructive diseases in the majority of rice-growing areas of the world [6]. Up to now, 27 ustilaginoidins have been reported from *V. virens* [7,8,9,10]. They showed teratogenicity to mouse embryo limb bud and midbrain cells, cytotoxicity toward human cancer cell lines, an inhibitory effect on mitochondrial respiration, phytotoxicity against the radicle and germ elongation of rice seeds, toxicity on zebrafish, and anti-bacterial activity [7,8,9,11,12]. Precise and sensitive determination of ustilaginoidins became an important requirement to meet food and feed safety concerns, as well as environmental security. Our research group has established HPLC–DAD and LC–MS to detect five main ustilaginoidins (i.e., ustilaginoidins A, B, C, G, and I) [9,13]. Although conventional instrument analysis is suitable for high-throughput screening of a large number of samples, it is expensive, time consuming, and labor intensive [14,15]. Immunoassay based on polyclonal antibody (pAb) or monoclonal antibody (mAb) have been proven to be a rapid and high-throughput method for monitoring mycotoxins in order to solve the disadvantages of instrument detection [16,17].

With the rapid development of molecular biology research for antibodies, increasingly more genetically engineered antibodies to detect small molecule compounds have been developed [18]. Recombinant antibodies such as fragment of antigen binding (Fab) and single-chain antibody fragment (ScFv) have been utilized in ELISAs and biosensors [19,20]. However, due to the low expression yield and stability of these antibody fragments, the application of this technique turns out to be problematic [21]. The naturally occurring antibodies without light chains are named heavy-chain antibodies and were found in 1993 in camels [22]. The variable domain of the heavy chain of heavy-chain antibody (VHH) was also called nanobody (Nb), having the advantages of high solubility, thermal stability, chemical stability, mass production, and easy editing. The elongated nanobody was thought to be easier to insert into narrow pits or grooves of a target substance [23]. Therefore, nanobodies have high utilization value and potential as an alternative reagent for next-generation immunoassays.

Owing to the excellent properties of nanobodies, many of them toward small molecules have been reported [24,25]. However, there are no nanobody studies regarding ustilaginoidins for rapid detection of ustilaginoidins in rice samples. In this study, an alpaca was immunized with hemiustilaginoidin F–H1–BSA, a VHH library was established, and five nanobodies were screened by phage display technology. The two types of nanobodies were further characterized for their selectivity and specificity using indirect competitive ELISA (ic–ELISA), and the molecular recognition mechanism between nanobodies and ustilaginoidins was primarily clarified.

## 2. Results

### 2.1. Construction of a Nanobody Library

**Figure 1:**
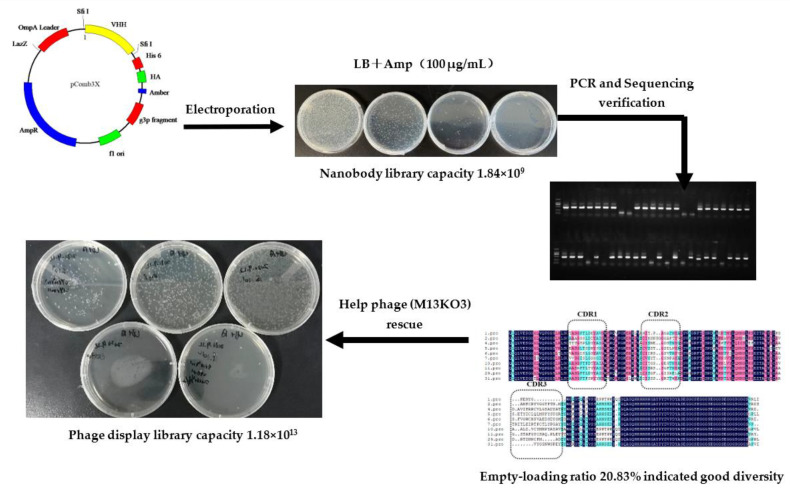
The construction process of nanobody phage library for ustilaginoidins in this study.

After the seventh immunization, the serum titer reached 3.2 × 10^4^, and the inhibition rate to ustilaginoidin A was 90% (Table S1), so 10 milliliters of blood was collected for total RNA extraction. RNA quality was measured by nanodrop and gel electrophoresis (Figure S1). After cDNA was obtained by reverse transcription, VHH genes were amplified by one-step PCR and ligated with phagemid pComb3X vector. The construction process of the library is shown in Figure 1. The ligated plasmid was transformed into *Escherichia coli* ER2738 by electroporation, and the nanobody library was estimated to have 1.84 × 10^9^ independent colonies. The established library was verified by colony-PCR with an empty load rate of 20.83%, and sequencing analysis showed that they had a good diversity. Finally, a nanobody phage display library with a titer of 1.18 × 10^13^ was obtained by helper phage rescue.

### 2.2. Selection of Phage Clones Speicific for Ustilaginoidins

**Figure 2:**
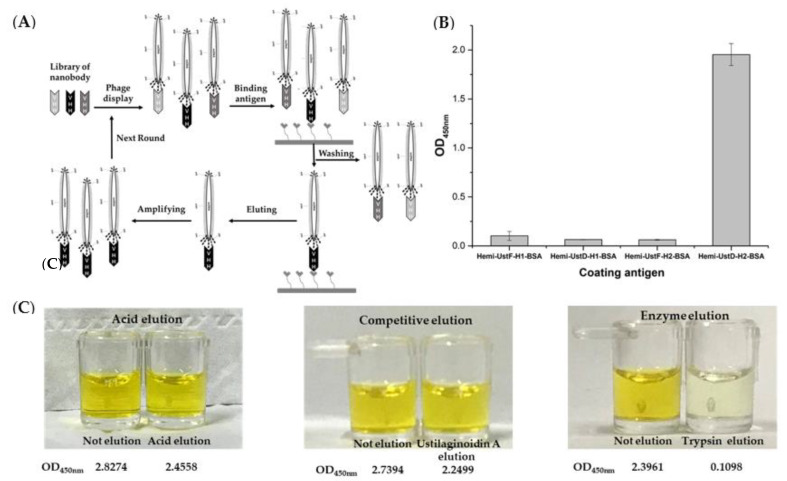
Optimization of nanobody phage display screening system. (**A**) Phage display selection cycle; (**B**) the effect of different coating antigen optimizations; (**C**) optimized effects of different elution methods. The data are the absorbance values at wavelength 450 nm.

The selection round for phage display illustrated in Figure 2A was carried out by referencing the previous method [26]. During the screening of the nanobody, the elution method and coating antigens were optimized. Hemiustilaginoidins F and D individually reacted with diazonium salt to obtain the haptens (i.e., hemiustilaginoidin F/D–H1), which further reacted with bovine serum albumin (BSA) to obtain their complete antigens (i.e., hemiustilaginoidin F/D–H1–BSA). At the same time, hemiustilaginoidins F and D individually reacted with formaldehyde and BSA to obtain hemiustilaginoidin F/D–H2–BSA. After the first round of screening, the supernatants of amplified phage were performed to polyclonal phage–ELISA in different coating antigens. The results showed that hemiustilaginoidin D–H2–BSA had the highest OD_450__nm_ value, which was close to 2, and the remaining coating antigens had almost no response (Figure 2B). When the immunogen (hemiustilaginoidin F–H1–BSA) and the coating antigen (hemiustilaginoidin D–H2–BSA) were in a heterologous form, the effect was found to be the best. In the process of phage display screening, the elution condition was also an important factor. The phage-ELISA method was used to detect the residual phage on the elution wells after being eluted by glycine (pH 2.2), ustilaginoidin A (1000 ng/mL), and trypsin (10 mg/mL). Figure 2C shows that the abilities of glycine elution and competitive elution were comparable. There was no significant color difference between eluted and un-eluted wells with around 85% of phage remaining in the eluted wells. When eluted with trypsin, the OD_450nm_ value in the eluted well was significantly lower than that of the un-eluted well, indicating that almost no phage remained in the eluted well with the strongest elution ability of trypsin.

On the basis of the results of three elution methods, a combination of trypsin and competitive elution was carried out (Table 1). The first round was eluted with trypsin, which had the strongest elution ability. At the second round, ustilaginoidin A (USA) at 1000 ng/mL was used for competitive elution, and then a more stringent elution of ustilaginoidin A/B/C (USA/USB/USC) at 200 ng/mL was carried out. Table 2 showed that with the increase in the selection pressure, the amount of output phages increased from 10^6^ to 10^7^ after each round of panning, indicating that the phages that specifically bind to hemiustilaginoidin D–H2–BSA were effectively enriched.

**Figure 3:**
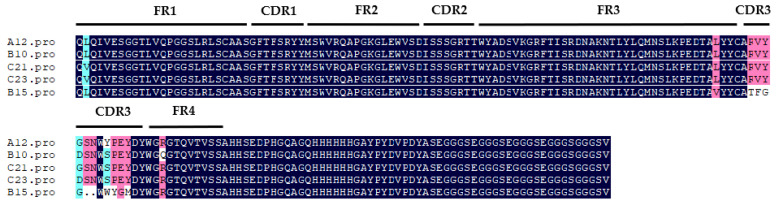
The amino acid sequences of five VHHs. FR1: Framework region 1; CDR1: complementarity-determining region 1; FR2: framework region 2; CDR2: complementarity-determining region 2; FR3: framework region 3; CDR3: complementarity-determining region 3; FR4: framework region 4.

Single clones were randomly picked on the output plate of each round for monoclonal phage-ELISA. Three wells were used for each monoclonal experiment; one well was set as the control well, in which only sample diluent was added. Two wells were set as the competition wells, one with 1000 ng/mL of USA and the other with 1000 ng/mL of USB. Monoclonal phages with 30% inhibition rate in two competing wells relative to the control well were judged as positive. Figure S2 shows that the primarily positive clones were as follows: 1–10, 1–11, 1–15, 1–17, 1–18, 1–19, 1–21, 1–22, 1–23, 1–24, A1, A2, A3, A6, A12, A17, A18, A20, B7, B10, B15, B16, B17, B19, B21, B23, B24, C10, C13, C16, C17, C20, C21, C23, J2, J6, J7, J9, J11, Y4, Y6, Y8, Y9, Y10, Y11, Y12, and Y13. The results of further sequencing analysis showed that there were five VHHs, named A12, B10, B15, C21, and C23 (Figure 3). Among them, the CDR3s of A12, B10, C21, and C23 were only one or two amino acids different from each other, which was classified as one type of nanobodies. Compared with the other four nanobodies, the CDR3 of B15 was significantly different, so B15 was individually classified as the other type of nanobody.

### 2.3. Expression and Verification of Nanobodies

**Figure 4:**
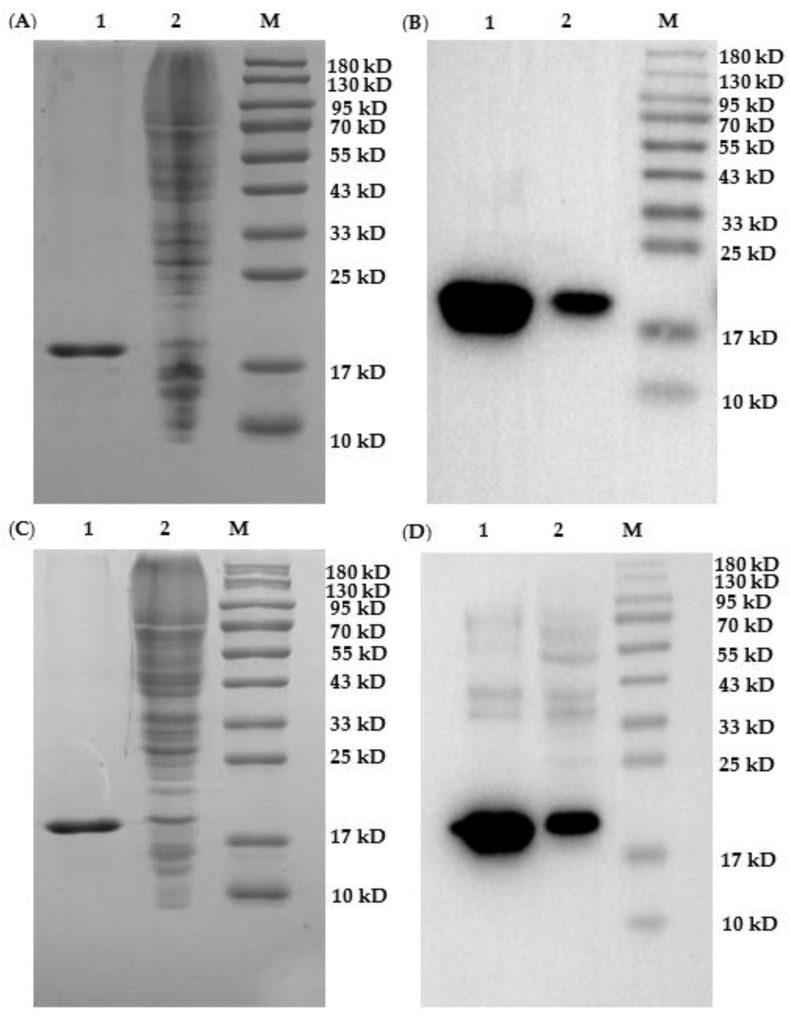
Identification of Nb–B15 and Nb–C21 expression. Analyses of SDS-PAGE (**A**) and Western blot (**B**) for Nb-B15; analyses of SDS-PAGE (**C**) and Western blot (**D**) for Nb–C21. Ladder 1: nanobody after purification on nickel column; Ladder 2: total protein extract after induction; M: marker.

Five nanobody vectors were transformed into non-suppressor *E. coli* strain Top10 cell for protein expression. During purification of nanobodies, 25 mM imidazole was used to elute the impurity protein, and 250 mM imidazole was used to elute the target protein. SDS-PAGE (Figure 4A,C and Figure S3C,E) showed that the purity of the target proteins (i.e., Nb–A12, Nb–B10, Nb-B15, Nb–C21, and Nb–C23) could reach more than 90% after expression purification on Ni−NTA affinity columns. As can be seen in Figure 4 and Figure S3, the size of the five nanobodies was about 17 kDa, which was consistent with the theoretical value. Furthermore, Western blot analysis (Figure 4B,D and Figure S3B,D,F) probing with HRP/anti-His also confirmed the successful expression of nanobodies. After lyophilization, the purification yields of Nb–A12, Nb-B10, Nb–B15, Nb–C21, and Nb–C23 were 3.75 mg/L, 4.12 mg/L, 5.50 mg/L, 5.20 mg/L, and 5.10 mg/L, respectively.

### 2.4. Nanobody ELISA

As the expressed nanobodies had two tags (i.e., HA-tag and His-tag), Nb–B15 was tested in three different forms (Figure S4A): HA-tag triple antibody method, His-tag triple antibody method, and His-tag dual antibody method. Figure S4B showed that under the competition of the same concentration of ustilaginoidin A, the inhibition rate of the HA-tag triple antibody method was significantly higher than those of the other two methods, reaching about 50%. Therefore, the HA-tag triple antibody method was used to establish the standard curve.

**Figure 5:**
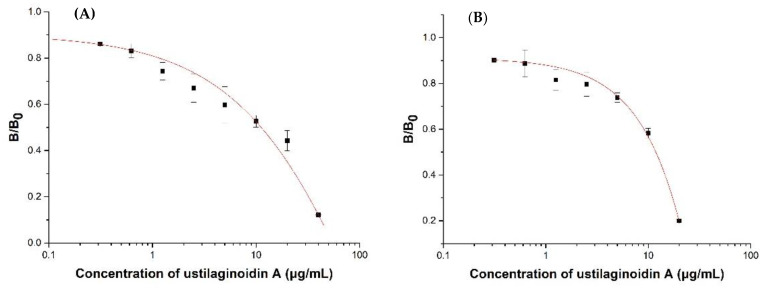
Standard inhibition curves of ustilaginoidin A in ic-ELISAs based on Nb–B15 (**A**) and Nb–21 (**B**). Each value represented the mean of triplicate ± standard deviation. B_0_ and B were the absorbance values at 450 nm in the absence and presence of ustilaginoidin A, respectively.

According to the protein expression yield and the titer results of the ELISA, Nb–B15 and Nb–C21 were selected as representatives of two types of nanobodies to draw standard inhibition curves (Figure 5). The concentration of coating antigen (hemiustilaginoidin D–H2–BSA) was 1 μg/mL, the Nb–B15/C21 concentration was 10 μg/mL, the HA-tag antibody concentration was 1.0 μg/mL, and the goat anti-mouse IgG-HRP was 1.0 μg/mL, determined by the checkerboard titration. The ic-ELISA standard curve based on Nb–B15 toward ustilaginoidin A is shown in Figure 5A; the IC_50_ value was 11.86 μg/mL, and the detection working range was 3.41–19.98 μg/mL. Figure 5B shows that for the ic-ELISA standard curve based on Nb–C21 for ustilaginoidin A, the IC_50_ value was 11.22 μg/mL, and the detection working range was 1.17–32.13 μg/mL.

### 2.5. Specificity of Nanobodies Nb–B15 and Nb–C21

The five main ustilaginoindins A, B, C, G, and I, along with other common mycotoxins such as aflatoxin B1, zearalenone, deoxynivalenol, ustiloxin A, and trichodimerol, were selected for cross-reactivity detection (Table 3 and Table 4). Nb–B15 had the strongest ability to recognize ustilaginoidin G with a cross-reactivity rate of 171.6%, and also to recognize ustilaginoidins B, C, and I with cross-reactivity rates of 30% to 80%.

**Figure 6:**
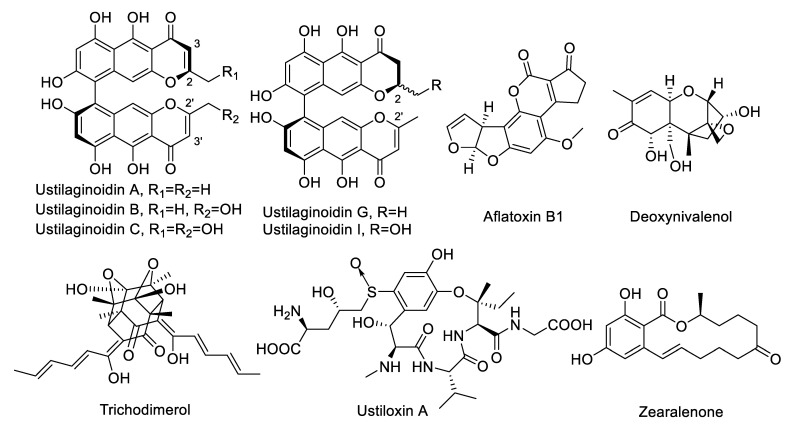
Structures of ustilaginoidins A, B, C, G, and I, and other mycotoxins tested in this study.

Hemiustilaginoidin F–H1–BSA was used for immunization, so the more similar the target compound was structurally related to hemiustilaginoidin F, the higher the recognition ability of nanobodies. Ustilaginoidins B and I were substituted with one hydroxymethyl group at C-2 or C-2′ by comparison with hemiustilaginoidin F (Figure 6), which affected the recognition of Nb–B15, so the cross-reactivity rates were 72.7% and 65.5%, respectively. There were two hydroxymethyl groups at C-2 and C-2′ of ustilaginoidin C in comparison with hemiustilaginoidin F, with only one methyl substitured at C-2 (Figure 6), so the cross-reaction rate was only 37.3%. Compared with ustilagnoidin A, ustilagnoidin G had no double bond between C-2 and C-3 (Figure 6), so Nb–B15 had a better ability to recognize ustilagnoidin G. The cross-reaction regulation of Nb–C21 was similar to that of Nb–B15 (Table 4).

### 2.6. Effects of Organic Solvents on Nb–B15 Binding Antigen

**Figure 7:**
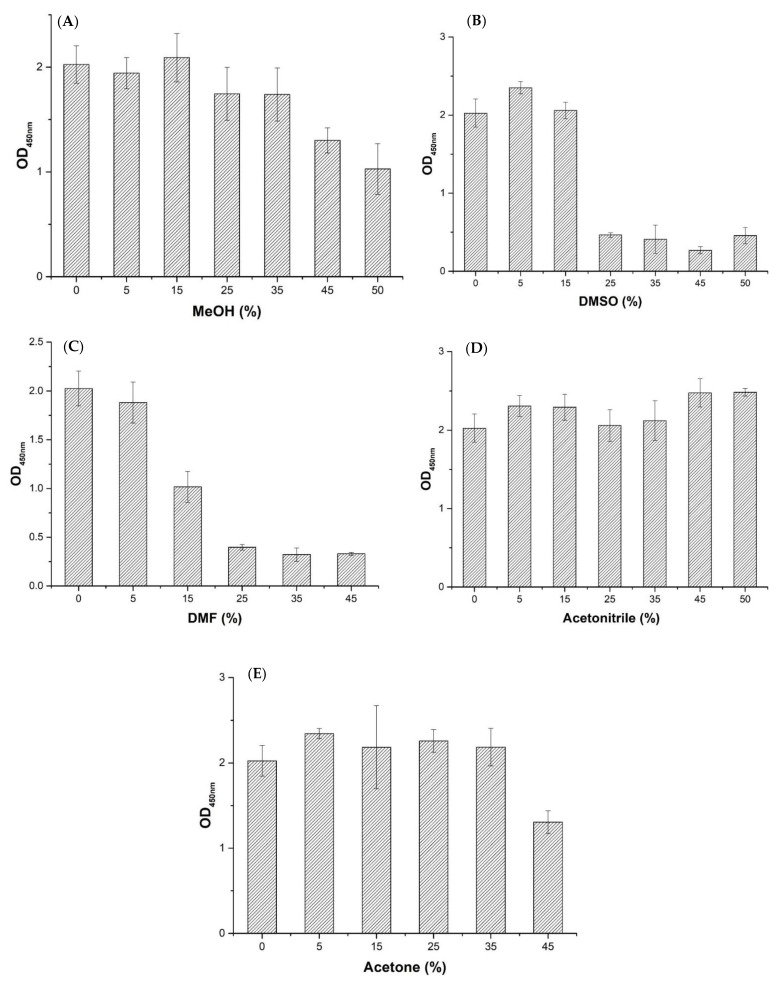
Effects of MeOH (**A**), DMSO (**B**), DMF (**C**), acetone (**D**), and acetonitrile (**E**) on the performance of Nb–B15-based ELISA. PBS buffers containing each organic solvent at different concentrations such as 10%, 30%, 50%, 70%, 90%, and 100% (*v*/*v*). The serial dilutions were mixed with equal volumes of Nb–B15, and then 100 μL of the mixture was added into hemiustilaginoidin D–H2–BSA–coated wells. The bound nanobodies were detected by adding 100 μL of anti HA-tag mAb for nanobody (1/1000 dilution in PBS) and goat anti-mouse-conjugated HRP (1/10,000 dilution in PBS), successively. TMB-chromogenic solution was added, and the absorbance values were read at a wavelength of 492 nm. Each value was the mean ± standard deviation of three well replicates.

Effects of five commonly used organic solvents such as methanol (MeOH), dimethyl sulfoxide (DMSO), *N*,*N*-dimethylformamide (DMF), acetonitrile, and acetone on Nb–15 binding antigen are shown in Figure 7. It shows that the Nb–B15 had a strong tolerance to methanol (MeOH), acetone, and acetonitrile. Among them, the tolerance of Nb–B15 gradually decreased as the concentration of methanol and acetone increased. However, Nb–B15 maintained more than 50% activity under the condition of 50% methanol and 45% acetone (Figure 7A,E). Moreover, the affinity of the Nb–B15 to ustilaginoidins was enhanced when treated with 50% acetonitrile (Figure 7D). It was found that Nb–B15 was intolerance in treatment with DMSO and DMF at 50% and 45%, respectively (Figure 7B,C). In the actual process of extracting ustilaginoidins, methanol and acetone were used, so the Nb-B15 could be applied to the detection of ustilaginoidin-contaminated samples. The influence of organic solvents for extraction in actual contaminative samples was also reduced. This property will also promote the development of immuno-affinity columns based on nanobodies, without high dilution of the samples extracted by methanol, acetone, or acetonitrile. Nb–B15 also showed strong tolerance at high temperature (Figure S7) wherein Nb–B15 retained 30% activity after 30 min treatment at 50 °C. In a pH tolerance test (Figure S8), Nb-B15 had good stability under neutral or acid conditions, but its stability decreased significantly in an alkaline environment.

### 2.7. Nb–B15-Based ELISA Analysis of Rice Samples

**Figure 8:**
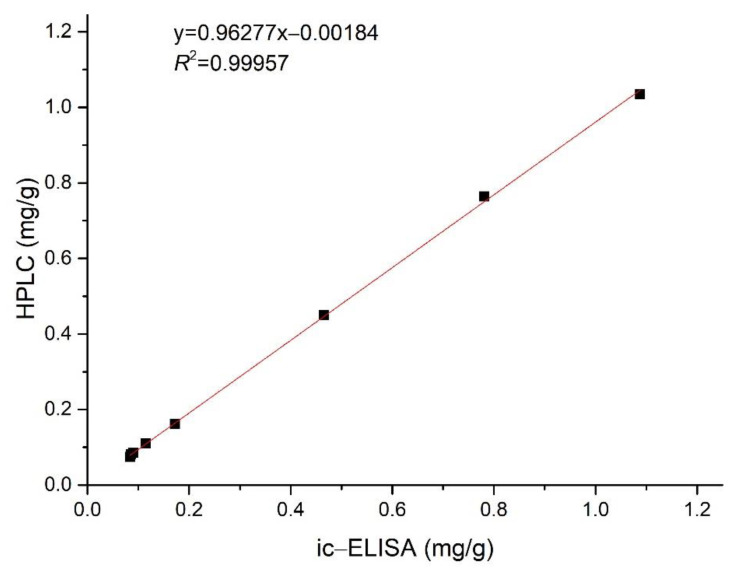
Correlation between ustilaginoidin content of the contaminated rice samples determined by ic−ELISA and by HPLC.

As Nb–B15 was superior to Nb–C21 in expression yield and titer, we chose Nb-B15 to detect actual rice samples. After rice samples were extracted with ethyl acetate, each extract was diluted with methanol for ELISA and HPLC analysis. The total amount of ustilaginoidins quantified by the ELISA analysis method was between 0.084 and 1.088 mg/g from contaminated samples collected from different areas (Table 5). In addition, there was no regular difference in the ustilaginoidin content of rice samples from different regions. The ic-ELISA results were always slightly larger than the HPLC test result as Nb–B15 could also recognize other trace ustilaginoidins in the contaminated samples, except the five main ustilaginoidins, which accounted for 95% of the total ustilaginoidins [13]. In general, the data obtained by ic-ELISA were in good agreement with the results of the HPLC. The correlation coefficient (*R^2^*) between the ic-ELISA and HPLC assays was 0.99957 (Figure 8). Therefore, the results demonstrated the reliability of ic-ELISA on the basis of Nb–B15 for ustilaginoidins. The developed method could be successfully employed to determine the content of total ustilaginoidins in contaminated rice samples.

### 2.8. Recognition Mechanisms of Nb–B15 and Nb–C21 against Ustilaginoidin A

The basic bioinformatic analyses of Nb–B15 and Nb–C21 were carried out. Both Nb–B15 and Nb–C21 belonged to hydrophilic proteins. By blast comparison, the similarity between the sequences of two nanobodies and 6HHU_H (PDB ID) reported in NCBI was 84.8%, and the homology was considered to be very high, so the structure of 6HHU_H was selected as the template to build the model. The quality of the model was evaluated by Ramachandran plot (Figure S5A,C) and ERRAT (Figure S5B,D). The Ramachandran plot of Nb–B15 (Figure S5A) showed that 85.0% of the amino acids of the model were located in the core region, 13.0% in the permissible region, 1.0% in the maximum permissible region, and only 1.0% in the forbidden region of torsion angle. ERRAT analysis showed that an overall quality factor of the Nb–B15 model was 91 (Figure S5B). The VERIFY-3D analysis showed that a VERIFY score in Nb–B15 model was 52.04, which was close to the VERIFY expected high score (53.7507). Thus, the Nb–B15 model was regarded to be of good quality. Similarly, 84.5% of the amino acids of the Nb–C21 model were in the core region, 13.6% in the permissible region, 1.0% in the maximum permissible region, and only 1.0% in the forbidden region of torsion angle from the Ramachandran plot of the Nb–C21 model (Figure S5C). ERRAT analysis (Figure S5D) showed that an overall quality factor of the Nb–C21 model was 88.496. VERIFY-3D analysis showed that the VERIFY score was 53.72, and the VERIFY expected high score was 54.6613, indicating that the Nb-C21 model was of good quality. Both created models could be used for subsequent docking research.

**Figure 9:**
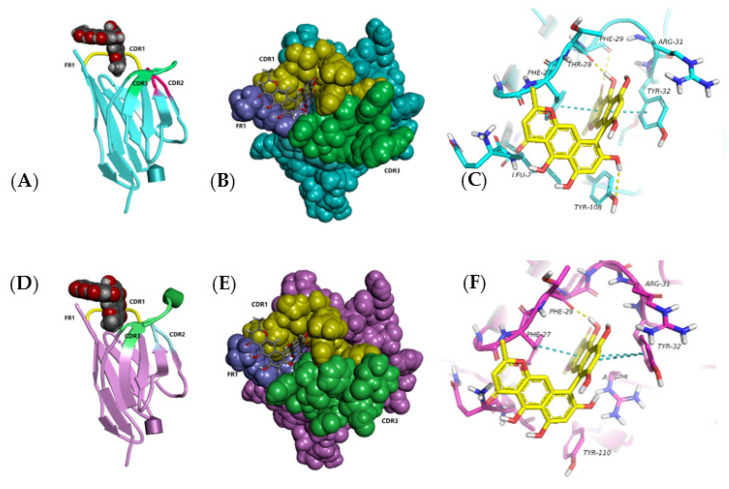
The recognition mode between Nb–B15 and Nb–C21 toward ustilaginoidin A in 3D docking plot. (**A**) Overall conformational docking analysis of Nb-B15 in cartoon style and ustilaginoidin A in sphere style. (**B**) Overall conformational docking analysis of Nb-B15 in sphere style and ustilaginoidin A in stick style. (**C**) The local docking analysis of Nb-B15 in stick style and ustilaginoidin A in stick style. The yellow dashed line represents the hydrogen bond interaction force, and the blue dashed line represents the pi–pi stacking interaction force. (**D**) Overall conformational docking analysis of Nb-C21 in cartoon style and ustilaginoidin A in sphere style. (**E**) Overall conformational docking analysis of Nb-C21 in sphere style and ustilaginoidin A in stick style. (**F**) The local docking analysis of Nb-C21 in stick style and ustilaginoidin A in stick style. The yellow dashed line represents the hydrogen bond interaction force, and the blue dashed line represents the pi–pi stacking interaction force.

Ustilaginoidin A was the most similar to hemiustilaginoidin F-H1 in structure and had the highest content in RFS, so we chose it as the ligand for molecular docking. The overall conformational analyses are shown in Figure 9A,B,D,E. Because the binding pocket was narrow and deep, it only accommodated the entry of one ring. One of the naphtho-γ-pyrone rings of ustilaginoidin A was inserted into the pocket formed by FR1, CDR1, and CDR3 of Nb–B15 or Nb–C21, and the other naphtho-γ-pyrone ring was exposed on the surface of the models. The position of 9,9′ connection of ustilaginoidin A was in at the exit of the pocket, and the exposed naphtho-γ-pyrone ring extends along the surfaces of CDR1 and FR1. From the local analysis of the docking between Nb–B15 and ustilaginoidin A (Figure 9C), Thr28 and Phe29 on Nb–B15 formed hydrogen bonds with the hydroxyl group at C-5 and the carbonyl group at C-4 on ustilaginoidin A, respectively. Tyr108 formed a hydrogen bond with the hydroxyl group at C-8′ on the ring of ustilaginoidin A exposed on the surface of models. The benzene rings of Phe27 and Tyr32 were simultaneously bonded to the six-membered ring of ustilaginoidin A to form a pi–pi stack interaction force. From the local analysis of the docking between Nb–C21 and ustilaginoidin A (Figure 9F), Phe29 on Nb–C21 formed a hydrogen bond with the hydroxyl group at C-5 on ustilaginoidin A. Tyr32 of Nb–C21 interacted with the two rings of the naphtho-γ-pyrone ring to form two pi–pi stack interaction forces, and Phe27 formed a pi–pi stack with the middle ring on the naphtho-γ-pyrone ring of ustilaginoidin A. In general, the key amino acid sites for the binding of Nb-B15 and Nb–C21 to ustilaginoidin A were mainly located in the FR1 and the CDR1 of two nanobodies. The differences between two nanobodies were those of amino acids in the CDR3 (Figure 3). Therefore, Nb-B15 and Nb–C21 had the same ability to recognize ustilaginoidin A theoretically. According to the ELISA experimental data (Figure 5) of the two nanobodies against ustilaginoidin A, their sensitivity to ustilaginoidin A was also very similar, which further proved the accuracy of the molecular docking results.

## 3. Discussion

### 3.1. Hapten Design

**Figure 10:**
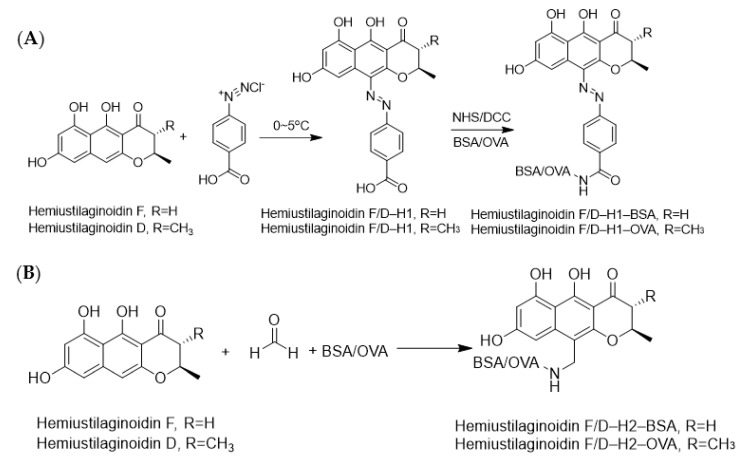
The conjugation of haptens with carrier proteins. (**A**) Synthesis routes of hemiustilaginoidin D/F-H1-BSA/OVA by diazotization reaction. (**B**) Synthesis routes of hemiustilaginoidin D/F-H2-BSA/OVA by Mannich reaction.

It is well known that immunogenicity could be acquired when a hapten was coupled with a macromolecule carrier [27]. For ustilaginoidins, both the ketone carbonyl group and the phenolic hydroxyl group were considered as the candidates to be modified. However, it was difficult for ustilginoidins to directly connect protein due to the large steric hindrance effect, so ustilaginoidins needed to be structurally modified. We tried to utilize monomeric naphtho-γ-pyrones or a part of ustilaginoidins to perform structural derivatization. Ustilaginoidins belonged to bis-naphtho-γ-pyrones, which were composed of two structurally similar naphtho-γ-pyrones. However, there was no report on the chemical synthesis of monomeric naphtho-γ-pyrones. It was found that two monomeric naphtha-γ-pyrones, namely, hemiustilaginoidins D and F, were obtained from the laccase gene deletion mutant of *V. virens* [28,29]. The precursor hemiustilaginoidin D/F of ustilaginoidins was used as the raw material for hapten synthesis through diazotization and Mannich reactions, followed by bioconjugation with carrier proteins for complete antigen synthesis (Figure 10).

### 3.2. Nanobody Screening

Specific nanobodies were commonly selected from the constructed nanobody libraries, which could be mainly classified into immune library, naïve library, and semisynthetic/synthetic library. Among them, the immune library was the most widely used strategy for nanobody screening [30]. In the screening process of the nanobody recognizing ustilaginoidins, an alpaca was immunized by hemiustilaginoidin F-H1-BSA to obtain the immune library. Phage display, ribosome display, yeast display, and bacteria display were often used for nanobody screening [30]. Antibody phage display libraries were the most widely applied to screen nanobody recognizing small molecules [25]. Smith firstly introduced the concept of displaying exogenous proteins on the surface of M13 phages in 1985 [31]. During phage display screening, coating antigens and elution method were optimized in this research. When the immunogen and the coating antigen were in a heterologous form, the screening effect was considered to be the best (Figure 2B). This phenomenon was also found in the screening of carbaryl and tetrabromobisphenol A nanobodies [32,33]. It was possible that when heterologous antigens were coated, small molecules could more easily compete with the nanobody from coating antigen. In the process of phage display screening, elution condition was also an important factor. Among them, acid elution, competitive elution, and trypsin elution were considered as the three most commonly used elution methods [25]. Trypsin elution was more efficient than acid elution and competition elution (Figure 2C). Trypsin could destroy the carboxyl-terminal peptide chains of lysine and arginine so that phages that were bound on the coated antigen were completely released. Considering that acid elution might disrupt the structure of phage with nanobody, a combination of trypsin and competitive elution was carried out. Trypsin was used to completely elute phages bound to the coated antigen in the first round of screening, and then a more stringent competitive elution method was used in the subsequent panning process. The advantage of this method could more effectively reduce the loss of positive clones.

Normally, the nanobody-based ELISA shows less sensitivity than the monoclonal antibody [25]. In this study, the nanobody-based ELISA also showed lower sensitivities. As the total content of ustilaginoidins in actual contaminated rice samples is usually at a relatively high level, the immunoassay established in this study should be valid for rapid detection of ustilaginodins. In future research, the sensitivity of nanobodies for ustilaginoidin analysis could be improved by increasing the affinity of the nanobody to ustilaginoidins [34]. Another possibility is to change signal output modes of immunoassay by using luciferase [35] and nanoparticles [36] (i.e., chemiluminescence [37]).

### 3.3. Nanobody Expression

Nanobodies have been successfully expressed in a variety of expression systems of bacteria, fungi, insect cells, mammalian cells, and plants [38]. The yields of nanobodies expressed in bacteria in this study were not high, with only 3–6 mg/L, but we could try to replace the expression vector or host to further improve their yields and activities in the future. Plants were also one of the hosts for expressing nanobodies, because of their features such as easy transformation and scale-up; they also reduced safety issues and were capable of performing post-translational modifications [38]. It has been reported that a rice transgenic system could be used to express anti-tumor necrosis factor alpha (TNF) nanobodies with an expression yield of 14.5 mg/g antibody/dry seed weight [39], indicating that the method of expressing nanobodies in rice was feasible. The nanobodies developed in this study could recognize ustilaginoidins produced from rice false smut pathogen *V. virens*. Therefore, the research that nanobody genes were transformed into rice to produce a toxin-neutralizing antibody for prevention of rice false smut will be worth studying in the future.

### 3.4. Molecular Recognition Mechanism

The recognition between antigen and antibody directly determined the performance of the detection method in immunoassays. The accuracy of the three-dimensional structure of the antibody is particularly critical for analyzing the interaction between antibody and antigen. At present, crystallization is the most accurate method for antibody structure, but there are problems such as high cost and harsh experimental requirements [40]. In recent years, computer simulation has increasingly become the means to obtain antibody structure [41]. For example, the recognition of the four key amino acids of nanobody toward ochratoxin A was verified by homology modeling, molecular docking, and alanine scanning. Two site-directed saturated mutation libraries were constructed by two-site mutation against those four key amino acids, and nanobodies with improved sensitivity were obtained [34]. In this research, we also identified four to five key amino acids, and a mutant library of several amino acids will be established to screen for more sensitive nanobodies in future research.

Several crystal structures of complexes of small molecules and nanobodies have been reported such as cortisol [42], methotrexate [43], red dye RP1 [44], red dye RP6 [45], triclocarban [46], and caffeine [47]. Among them, the crystal structure of the complex of caffeine and nanobody was the most similar to the recognition mechanism of the nanobody against ustilaginoidin A in this study. Tyr34 and Tyr104 on each nanobody directly interacted with caffeine through hydrogen bonding and pi–pi stacking, respectively, in order to form dimers from the crystal structure (Figure S6). Ustilaginoidin A was more structurally symmetrical than caffeine. From the overall conformational analyses (Figure 9A,B,D,E), one of the naphtho-γ-pyrone rings of ustilaginoidin A was inserted into the pocket formed by FR1, CDR1, and CDR3 of Nb-B15 and Nb-C21, and the other loop did not enter the pocket. The naphtho-γ-pyrone ring exposed on the surface was likely to be linked with the other nanobody to form a dimer. This hypothesis needs to be further verified by obtaining the crystal structure.

## 4. Conclusions

In this research, the precursor hemiustilaginoidin D/F of ustilaginoidins was used as the raw material for hapten synthesis through diazotization and Mannich reactions, and then bioconjugation with carrier protein for synthesis of a complete antigen (i.e., hemiustilaginoidin D/F-H1-BSA/OVA and hemiustilaginoidin D/F-H2-BSA/OVA). This is the first time that a specific hapten has been obtained by combining methods of gene deletion and chemical synthesis. An alpaca was immunized with hemiustilaginoidin F-H1-BSA, and nanobodies that recognized ustilaginoidins were firstly obtained by screening from a nanobody phage display library. A phage display screening strategy for ustilaginoidins was established by optimization, mainly including elution mode and coating mode. Both trypsin elution and competition elution were alternately performed, and the immunogen and the coating antigen were heterologous for successful screening. Five nanobodies were successfully screened, and they were divided into two types according to their amino acid sequences. Two nanobodies, Nb–B15 and Nb–C21, were selected for the establishment of ic-ELISA.

On the basis of Nb–B15, the IC_50_ value was 11.86 μg/mL and the detection range was 3.41–19.98 μg/mL in ic-ELISA. On the basis of Nb–C21, the IC_50_ value was 11.22 μg/mL and the detection range was 1.17–32.13 μg/mL in ic-ELISA. The nanobody-based ELISA achieved in this study was less sensitive and needs to be improved in future research. Both Nb-B15 and Nb–C21 were able to recognize the main five ustilaginoidins with a broad spectrum according to cross-reactivity to quantify the content of total ustilaginoidins. Molecular recognition mechanisms between Nb–B15/C21 and ustilaginoidin A were elucidated from the overall conformation and local conformation through homology modeling and molecular docking. In general, a nanobody-based immunoassay method for analysis of total ustilaginodins in rice samples was developed. It will provide a rapid and effective strategy for detection of total ustilaginoidins in contaminated rice samples and any other environmental samples. It also has potential applications in the field of biotechnology such as screening of fungal mutants with a high yield of ustilaginoidins, as well as in rice RFS-resistance breeding.

## 5. Materials and Methods

### 5.1. Chemicals and Reagents

Trypsin and yeast extract were purchased from OXOID (Oxoid Ltd., Basingstoke, Hampshire, UK). Bovine serum albumin (BSA), ovalbumin (OVA), Tween 20, 3,3′,5,5′-tetramethylbenzidine (TMB), isopropyl-β-D-thiogalactopyranoside (IPTG), imidazole, Freund’s complete adjuvant, Freund’s incomplete adjuvant, horseradish-peroxidase-labeled anti-M13 mouse monoclonal antibody, ampicillin sodium salt (Amp), kanamycin sulfate (Kana), tetracycline hydrochloride (Tetr), and TOP10F′ cells were purchased from Sigma-Aldrich (St. Louis, MO, USA). B-PER™ bacterial protein extraction reagent, protein molecular weight marker, and reverse transcription kit were purchased from Thermo Fisher Scientific Inc. (Rockford, IL, USA). Complete His-Tag Purification Resin was purchased from Roche Pharmaceutical Co., Ltd. in Shanghai, China. Anti-HA tag and anti-His tag mouse monoclonal antibodies were homemade in the laboratory. Phagemid vector pComb3X was a generous gift from Dr. Carlos F. Barbas (The Scripps Research Institute, La Jolla, CA, USA). QIAprep Spin MiniPrep Kit, QIAquick Gel Extraction Kit, and QIAquick PCR Purification Kit were all from Qiagen (Qiagen Ltd., Hilden, Germany). The Total RNA extraction kit was bought from Magen Bio (Magen Biotechnology Co., Ltd., Guangzhou, China).

### 5.2. Preparation of Immunogen and Coating Antigens

Both diazotization and Mannich reactions were used to synthesize immunogens and coating antigens (Figure 10). Hemiustilaginoidin D/F–H1–BSA/OVA indicated complete antigen synthesized by diazo reaction [48]. Hemiustilaginoidin D/F was coupled to BSA/OVA via Mannich reaction [49,50]. A total of 4.2 mg of hemiustilaginoidin D/F was dissolved in 1 mL of DMF, which was divided into two equal parts into vials, and then 3.5 mg BSA and 2.5 mg OVA water solution were added, respectively. After the mixture was stirred at room temperature for 5 min, 50 μL of coating buffer and 60 μL of 30% formaldehyde were added into each vial, and the mixture was stirred at room temperature overnight. The entire reaction solution was transferred into dialysis bags, at pH 7.5, 0.1 M PBS buffer for 6 times, 6 h for each time, and then obtain hemiustilaginoidin D/F–H2–BSA/OVA with 1 mg/mL based on the weight of BSA or OVA.

### 5.3. Alpaca Immunization and Antiserum Assessment

A 2-year-old male alpaca was immunized subcutaneously with 200 μg hemiustilaginoidin F–H1–BSA with the same volume of Freund’s complete adjuvant at the first injection. The following injections were performed every 2 weeks with Freund’s incomplete adjuvant. Before the first immunization, 5 mL of blood was taken as blank control. Following this, 20 mL of blood was collected on day 7 after each immunization from fifth to seventh immunization. Then, 1 mL blood was reserved for antiserum assessment, and 19 mL blood was used for the isolation of leukocytes. Antiserum assessment steps were as follows. In brief, a 96-well microplate (Corning, NY, USA) was coated with hemiustilaginoidin F–H1–OVA in carbonate buffer at 100 μL per well at 37 °C for 3 h. The plate was washed with PBST (0.2 g/L KH_2_PO_4_, 8.0 g/L NaCl, 2.96 g/L Na_2_HPO_4_ × 12H_2_O, and 1% Tween-20) four times. A total of 50 μL of ustilaginoidin A (1000 ng/mL) in PBSTG (PBST comprising 1 g/L gelatin) was pipetted into each well, followed by an addition of 50 μL of sera solution diluted in PBSTG. After incubation at 37 °C for 0.5 h, the plate was washed four times to remove the unbound antibodies, and 100 μL per well of goat anti-alpaca-HRP (1 μg/mL) in PBSTG was added. The plate was incubated at 37 °C for 30 min and then washed again with PBST, as mentioned above. Finally, 100 μL of TMB-chromogenic solution (5 mL of TMB storage solution and 5 mL of substrate buffer, and 10 μL of 30% H_2_O_2_) was added into each well. The reaction was terminated by adding 50 μL of 2 M H_2_SO_4_ per well after 10 min. Absorbance was read at 450 nm on a Multiskan MK3 microplate reader (Thermo, Vantaa, Finland).

### 5.4. Phage-Displayed Library Construction

According to the titer and inhibition by ustilaginoidin A of alpaca serum (Supplementary Material Table S1), the blood was collected for RNA extraction and library construction after the seventh immunization. The library was constructed according to the method of Kim et al. [51]. RNA was extracted from blood and was then transcripted to cDNA according to the kit instructions. By referencing the method of He et al. [52], the nanobody gene fragments from IgG2 and IgG3 were amplified by PCR using two pairs of primers: AlpVHH-R1 *SfiI* (CATGCCATGACTCGCGGCCGGCCTGGCCATGGGGGTCTTCGCTGTGGTGCG) and Alp-VHH-F1 *SfiI* (CATGCCATGACTGTGGCCCAGGCGGCCCAGKTGCAGCTCGTGGAGTC); AlpVHH-R2 *SfiI* (CATGCCATGACTCGCGGCCGGCCTGGCCGTCTTGTGGTTTTGGTGTCTTGGG), and Alp-VHH-F1 *SfiI*. The PCR products and pComb3x phagemid vector were separately digested with SfiI and subsequently ligated to generate pComb3x/nanobody constructs. After electroporation of the target genes into *Escherichia coli* ER2738, the library size was estimated by counting clones on LB agar plates containing ampicillin at 100 mg/L. A total of 48 single clones were verified by PCR to calculate the library empty load rate. Ten clones selected from PCR-positive clones were sequenced to evaluate library diversity using g-back primer (GCCCCCTTATTAGCGTTTGCCATC). The library was rescued by helper phage to obtain a nanobody phage-display library.

### 5.5. Biopanning and Nanobody Screening

One day before screening, 500 μL of the original phage library or previous round phage library was premixed with an equal volume of 2% BSA/PBS solution and placed at 4 °C overnight. Hemi–UstD–H2–BSA and BSA (10 μg/mL) were coated on a strip of eight wells on the ELISA plate, individually, overnight at 4 °C. The next day, the coated plate was washed 5 times with PBST. The premixed phage solution was added to the BSA-coated wells, and 200 μL of blocking solution was added to the hemi-UstD-H2-BSA wells. Both were placed in a 37 °C incubator for 1 h. The blocking solution in the Hemi–UstD-H2–BSA wells was dried and then washed with PBST. A total of 100 μL of the phage library was pipetted from BSA wells into the Hemi–UstD–H2–BSA wells, then was shaken for 2 h at room temperature, dried vigorously, and washed with PBST. Subsequently, trypsin (10 mg/mL) or ustilaginoidins standard was added to the wells for elution, and eluted phages of each round were used as the output of this round. After the expansion culture, it was used as the input library for the next round of panning. The phage enrichment was observed by calculating the phage titer before and after each round of panning. The panning conditions of different rounds are shown in Table 1.

### 5.6. Polyclonal and Monoclonal Phage-ELISA

After three rounds of panning, 96 clones were selected from each round output titer plate and amplified each in 3 mL of SB medium (10 g/L yeast extract, 10 g/L tryptone, 5 g/L NaCl). After overnight cultivation, the culture was centrifuged at 3000× g for 5 min, and the supernatant was further characterized by monoclonal phage-ELISA. The amplified phages were centrifuged to take the supernatant for polyclonal phage-ELISA after each round of screening. The phage-ELISA procedure is as follows: hemiustilaginoidin D-H2-BSA (1 μg/mL) was coated overnight at 4 °C, and 50 μL USA (1000 ng/mL), 50 μL USB (1000 ng/mL), and 50 μL blank control were separately added to three wells. Then, 50 μL phage supernatant was added. After incubation at 37 °C for 0.5 h, the plate was washed four times to remove the unbound phage, and 100 μL per well of anti-M13-HRP mouse mAb (1 μg/mL) in PBSTG (PBST comprising 1 g/L gelatin) was added. The following steps were the same as Section 3.3 (Alpaba Immunization and Antiserum Assessment). The phage clones with inhibition rate as 30% in two competing wells relative to blank control wells were judged as being positive.

### 5.7. Expression and Purification of Five Nanobodies

Phagemids from the five positive clones, namely, Nb–A12, Nb–B10, Nb–B15, Nb–C21, and Nb–C23, were transformed into *E. coli* strain TOP10F′ cells. Nanobody expression was performed after PCR validation and sequencing analysis to verify successful transformants. A total of 200 mL of SB medium was incubated with an overnight culture of TOP10F′ cells carrying nanobody expression plasmid and incubated at 37 °C with shaking at 250 rpm. When the culture reached an OD_600nm_ value of 0.6–0.8, 1 mM IPTG was added, followed by continuous shaking overnight. The bacteria were collected by centrifugation. After extraction of the nanobody from periplasmic space by B-PER™ bacterial protein extraction reagent, the nanobody with 6× His tag and HA-tag at C-terminal was purified with a Ni-NTA column. When nanobodies were purified, 25 mM imidazole buffer was used to elute impurity proteins, and 250 mM imidazole was used to elute target proteins. Then, through dialysis and freeze-drying treatment, nanobody powder was obtained, which was stored at −80 °C for later use. The purity and size of nanobodies were assessed using 15% reducing SDS-PAGE and Western blot according to a standard protocol, followed by staining with Coomassie Brilliant Blue and eECL Western Blot Kit, respectively.

### 5.8. Preparation and Detection of Ustilaginoidins of Rice Samples

Rice samples were extracted in accordance with the previous method [13]. Briefly, each rice sample (100 mg) was weighed and then extracted with ethyl acetate (EtOAc) three times (3 × 1 mL, 30 min for each time) in an ultrasonic bath at room temperature. The EtOAc extract was concentrated by a rotary evaporator to dryness under vacuum at 28 °C. The obtained residue was dissolved in 1 mL of MeOH and was diluted 100 times by PBSTG, then was added into a microplate for ic-ELISA.

Each sample was filtered through a microporous filter (pore size, 0.22 μm) before HPLC analysis. For HPLC analysis, each solution was filtered and analyzed by an HPLC system eluted with a linear gradient of methanol from 50 to 100% (*v*/*v*) and water (containing 0.01% oxalic acid) from 50 to 0% (*v*/*v*) over 40 min at a flow rate of 1.0 mL/min. The temperature was maintained at 30 °C and UV detection was maintained at 290 nm; moreover, the sample injection volume was at 10 μL. The analytical Prominence LC-20A HPLC instrument was employed to acquire and process chromatographic data according to the method of Meng et al. [13]. Through quantification of each ustilaginoidin (i.e., ustilaginoidins A, B, C, G, and I) content, the sum of the five ustilaginoidins was estimated as the total content of ustilaginoidins in the samples.

### 5.9. Modeling and Docking

The basic bioinformatics analysis of Nb–B15 and Nb–C21 was analyzed by the ExPASy Protparam database. The homology modeling was carried out using Modeller 9.21 with the most similar template selected through the Blast alignment of NCBI. The quality of the model was evaluated by the Structure Analysis and Verification Server (SAVES, https://services.mbi.ucla.edu/SAVES/, accessed on 30 November 2021) and through the use of Discovery Studio (Accelrys Ltd., San Diego, CA, USA). The established model proteins were pretreated with Maestro 12.0. The binding pocket of the protein was predicted using the sitemap in Maestro 12.0, and the pocket radius was adjusted according to the spatial conformation of the CDR region of the nanobody. Thus, it could cover all theoretically possible binding sites. The ustilaginoidin A molecule was constructed by ChemDraw 2019 (Cambridge Soft Ltd., Waltham, MA, USA) and saved in sdf format, and it was pretreated with ligprep in Maestro12.0 to rationalize its structure. After treatments of the model and ustilaginoidin A, a docking grid file was generated by Maestro 12.0; Glid was used for docking, and the docking mode was SP. According to the score, the docking result with the best conformation was selected. Interaction force analysis and graphing were performed using Pymol 1.6.5 software (Schrodinger Ltd., Milford on Sea, UK).

## Figures and Tables

**Table 1:** Panning strategy of phage display for screening nanobodies against ustilaginoidins.

Round	Elution Method	Washing Times
1	Trypsin elution	10
2	Trypsin and competitive elution (USA, 1000 ng/mL)	15
3	Competitive elution (USA/USB/USC, 200 ng/mL)	20

Note: USA, ustilaginoidin A; USB, ustilagidoidin B; USC, ustilaginoidin C.

**Table 2:** Titers of input and output phages after each round of panning.

Round	Input (pfu/mL)	Output (pfu/mL)
1	1.18 × 10^13^	7.6 × 10^6^
2	1.5 × 10^13^	Trypsin 1.1 × 10^7^/competitive elution 9.9 × 10^6^
3	2.0 × 10^13^	USA 8.1 × 10^7^/USB 6.5 × 10^7^/USC 7.5 × 10^7^

Note: USA, ustilaginoidin A; USB, ustilagidoidin B; USC, ustilaginoidin C. Input indicates the amount of input phage per milliliter. Output indicates the amount of output phage per milliliter.

**Table 3:** Cross-reactivities of Nb–B15 with other compounds.

Analyte	IC_50_ (μg/mL)	Cross-Reactivity (%)
Ustilaginoidin A	11.86 ± 0.53	100 ± 4.4
Ustilaginoidin B	16.31 ± 0.91	72.7 ± 7.7
Ustilaginoidin C	31.83 ± 1.49	37.3 ± 1.7
Ustilaginoidin G	6.95 ± 1.43	171.6 ± 29.6
Ustilaginoidin I	18.11 ± 0.37	65.5 ± 1.4
Aflatoxin B1	ND	ND
Zearalenone	ND	ND
Deoxynivalenol	ND	ND
Trichodimerol	ND	ND
Ustiloxin A	ND	ND

Note: ND means not detected. Data represent means of triplicate ± standard deviations.

**Table 4:** Cross-reactivities of Nb–C21 with other compounds.

Analyte	IC_50_ (μg/mL)	Cross-Reactivity (%)
Ustilaginoidin A	11.22 ± 0.73	100 ± 4.5
Ustilaginoidin B	12.90 ± 0.77	86.9 ± 5.6
Ustilaginoidin C	32.00 ± 4.31	35.0 ± 4.5
Ustilaginoidin G	12.06 ± 2.08	125.0 ± 16.9
Ustilaginoidin I	16.04 ± 2.31	69.9 ± 7.2
Aflatoxin B1	ND	ND
Zearalenone	ND	ND
Deoxynivalenol	ND	ND
Trichodimerol	ND	ND
Ustiloxin A	ND	ND

Note: ND means not detected. Data represent means of triplicate ± standard deviations.

**Table 5:** Comparison of Nb–B15-based ELISA and HPLC analysis of total ustilaginoidins in contaminated samples collected from different areas of China.

Collection Area (Longitude, Latitude, Time)	Nb–B15-Based ELISA (mg/g)	HPLC (mg/g)
Chengdu (104.1° E, 30.6° N), Sichuan, China, in 2014	0.115 ± 0.002	0.110 ± 0.009
Hefei (117.2° E, 31.8° N), Anhui, China, in 2014	1.088 ± 0.020	1.035 ± 0.074
Guilin (110.7° E, 25.6° N), Guangxi, China, in 2015	0.781 ± 0.067	0.764 ± 0.019
Anqing (116.6° E, 30.6° N), Anhui, China, in 2017	0.465 ± 0.013	0.449 ± 0.059
Donggang (124.2° E, 39.9° N), Liaoning, China, in 2017	0.090 ± 0.006	0.085 ± 0.005
Haidian (116.2° E, 40.1° N), Beijing, China, in 2018	0.084 ± 0.006	0.074 ± 0.007
Kaili (107.5° E, 26.3° N), Guizhou, China, in 2018	0.086 ± 0.004	0.081 ± 0.013
Enshi (109.3° E, 30.2° N), Hubei, China, in 2019	0.172 ± 0.014	0.161 ± 0.009

Note: The result of ic-ELISA was the content of total ustilaginoidins; the result of HPLC was the sum of the contents of five main ustilaginoidins. Data represent means of triplicate ± standard deviations.

## Data Availability

Not applicable.

## Supplementary Materials

The following supporting information can be downloaded at https://www.mdpi.com/article/10.3390/toxins14100659/s1, Figure S1: Gel electrophoresis of total RNA from the alpaca. Figure S2: Identification of the positive phage clones from each round of panning. Figure S3: Identification of Nb–A12, Nb–B10, and Nb–C23 expression. Figure S4: Schematic detections with different ic-ELISAs and their inhibition rates. Figure S5: Quality evaluation of Nb-B15 and Nb-C21 models. Figure S6: Complex of AcVHH dimer with caffeine. Figure S7: Thermo-stability of Nb-B15 treated at 50 °C in different time periods. Figure S8: Effects of different pH values on the performance of Nb-B15-based ELISA. Table S1: Titer and inhibition by ustilaginoidin A of alpaca serum after immunization. Reference [47] is cited in the Supplementary Materials.
