# Supplementary material for: A Nanobody-Based Immunoassay for Detection of Ustilaginoidins in Rice Samples
Source: Toxins (Basel). 2022 Sep 23;14(10):659. doi: 10.3390/toxins14100659 (PMC9609001; doi:10.3390/toxins14100659)
Supplement: Supplementary file 1 [file toxins-14-00659-s001.zip › toxins-1909386-supplementary.pdf]

# Supplementary Materials:

# A Nanobody-based Immunoassay for Detection of Ustilaginoidins in Rice Samples

## Contents

**Figure S1.** Gel electrophoresis of total RNA from alpaca. **Lane 1**: DNA marker. **Lane 2**: Total RNA.................... 2

**Figure S2.** Identification of the positive phage clones from each round of panning. **(A)** Phage clones (1-1~1-24) picked after the first round. **(B)** Phage clones (J-1~J-11) and phage clones (Y-1~Y-13) picked after competitive elution and trypsin of the second round respecticely. **(C)** Phage clones (A-1~A-24) picked after USA elution of the third round. **(D)** Phage clones (B-1~B-24) picked after USB elution of the third round. **(E)** Phage clones (C-1~C-24) picked after USC elution of the third round. ........................................................................................... 3

**Figure S3.** Identification of Nb-A12, Nb-B10 and Nb-C23 expression. Analysis of SDS-PAGE **(A)** and Western Blot **(B)** for Nb-A12; Analysis of SDS-PAGE **(C)** and Western Blot **(D)** for Nb-B10; Analysis of SDS-PAGE **(E)** and Western Blot **(F)** for Nb-C23; Ladder 1: Nanobody after purification on nickel column; Ladder 2: Total protein extract after induction; M: Marker.......................................................................................................................... 5

**Figure S4**. Schematic detections with different icELISAs and their inhibition rates. **(A)** Diagram of different detection forms; **(B)** Inhibition rates........................................................................................................... 6

**Figure S5.** Quality evaluation of Nb-B15 and Nb-C21 models. (**A**) Ramachandran plot of the Nb-B15 model; (**B**) ERRAT of the Nb-B15 model; (**C**) Ramachandran plot of the Nb-C21 model; (**D**) ERRAT of the Nb-C21 model. The asterisks (**) in (B) and (D) are expressed as the percentage of the protein for which the calculated error value falls below the 95% rejection limit. Good high resolution structures generally produce values around 95% or higher. For lower resolutions (2.5 to 3 Å), the average overall quality factor is around 91%......................... 7

**Figure S6**. Complex of AcVHH dimer with caffeine. The x-ray structure of acVHH dimer is in cartoon style, and caffeine is in orange/blue/red stick style [47]....................................................................................................... 7

**Figure S7.** Thermo-stability of Nb-B15 treated at 50 °C in different time. ........................................................ 8

**Figure S8.** Effects of different pH values on the performance of Nb-B15-based ELISA. .................................... 8

**Table S1.** Titer and inhibition by ustilaginoidin A of alpaca serum after immunization. .................................. 9

**Supplementary References** ......................................................................................................................... 9

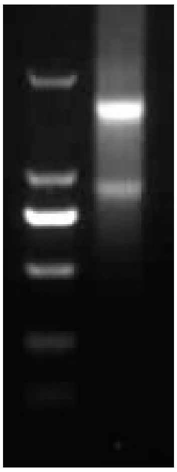


**Figure S1.** Gel electrophoresis of total RNA from alpaca. **Lane 1**: DNA marker. **Lane 2**: Total RNA.

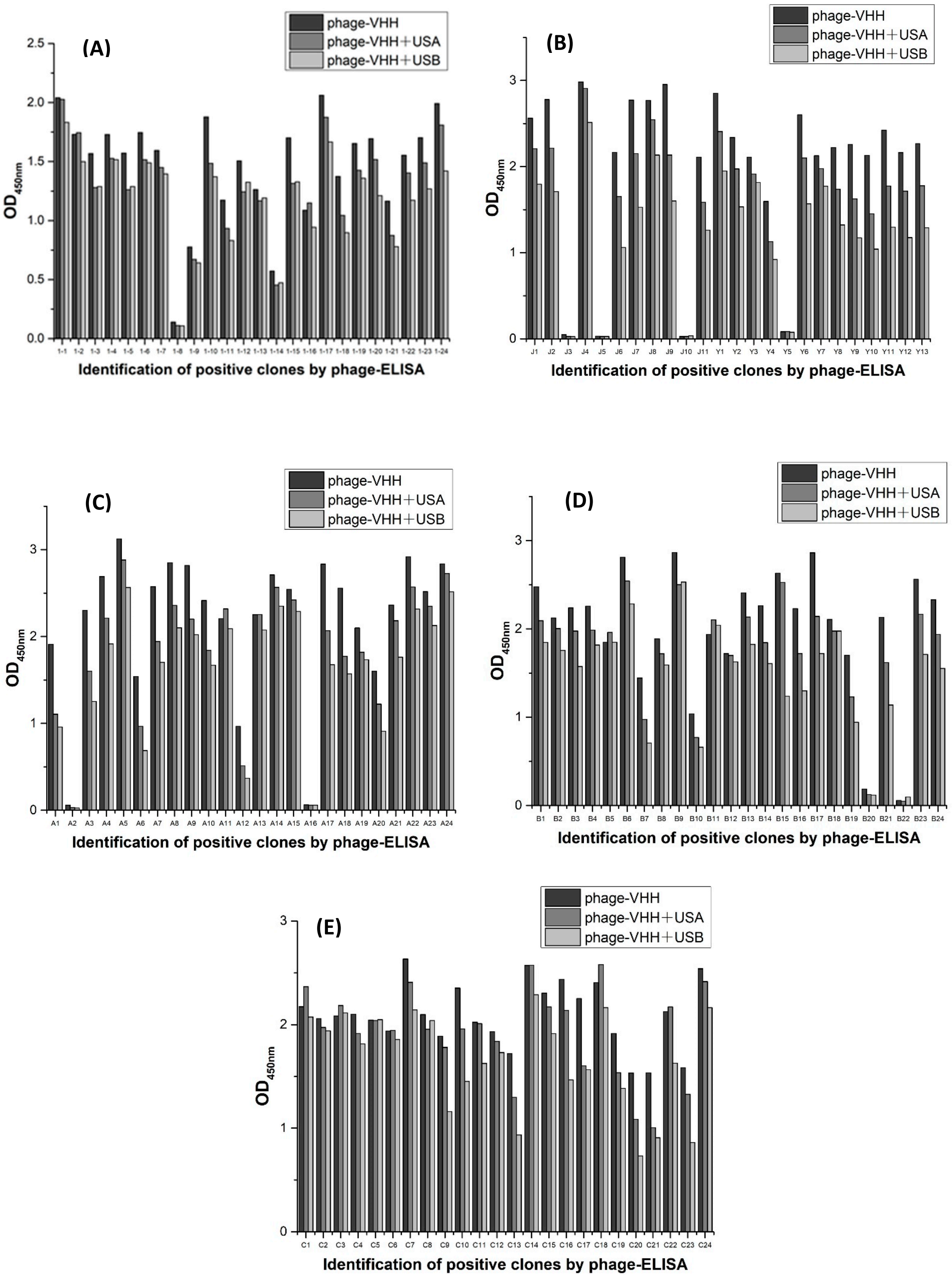


**Figure S2.** Identification of the positive phage clones from each round of panning. **(A)** Phage clones (1-1~1-24) picked after the first round. **(B)** Phage clones (J-1~J-11) and phage clones (Y-1~Y-13) picked after competitive elution and trypsin of the second round respecticely. **(C)** Phage clones (A-1~A-24) picked after USA elution of the third round. **(D)** Phage clones (B-1~B-24) picked after USB elution of the third round. **(E)** Phage clones (C-1~C-24) picked after USC elution of the third round.

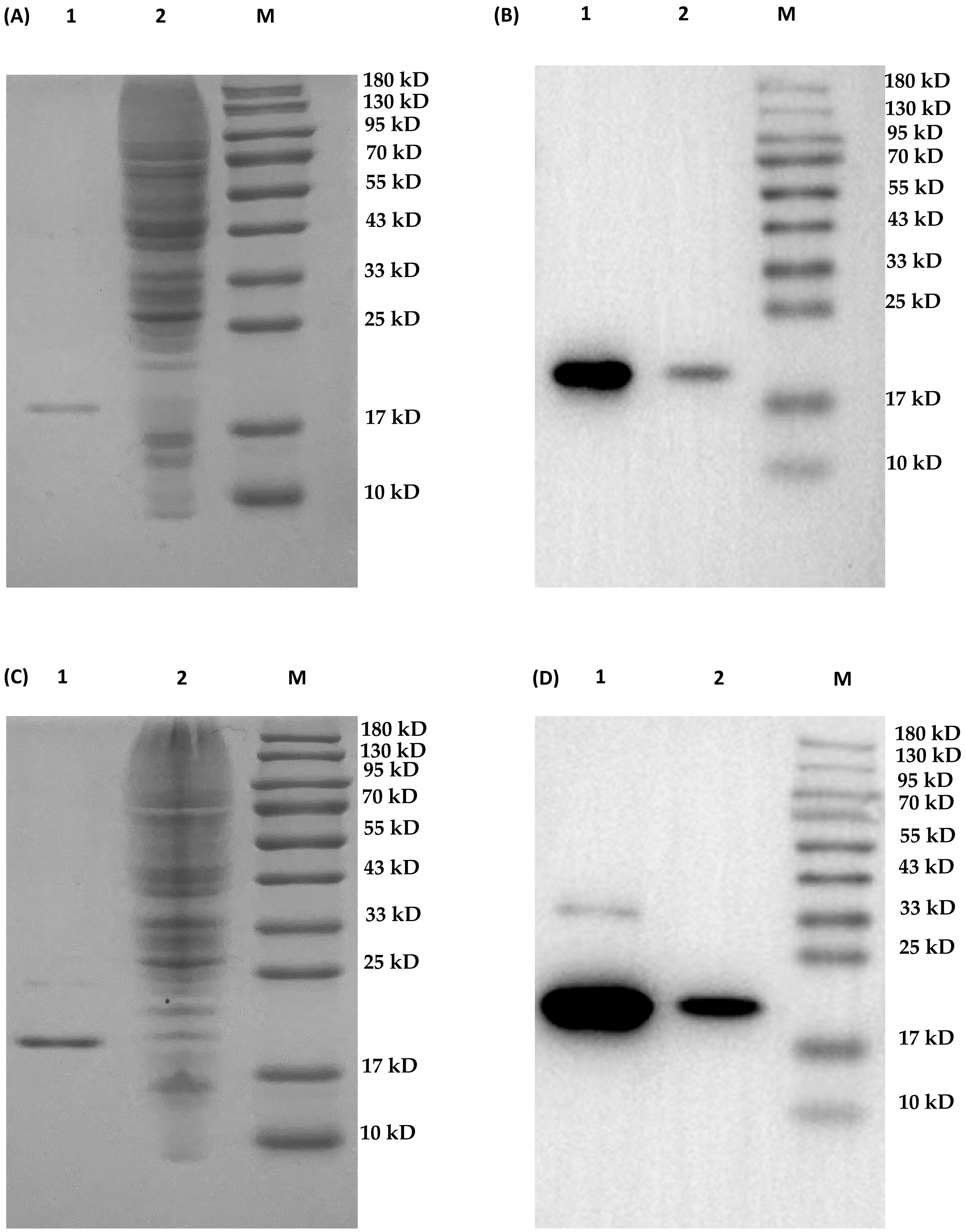
(A)
1
2
M
180 kD
130 kD
95 kD
70 kD
55 kD
43 kD
33 kD
25 kD
17 kD
10 kD
(B)
1
2
M
180 kD
130 kD
95 kD
70 kD
55 kD
43 kD
33 kD
25 kD
17 kD
10 kD
(C)
1
2
M
180 kD
130 kD
95 kD
70 kD
55 kD
43 kD
33 kD
25 kD
17 kD
10 kD
(D)
1
2
M
180 kD
130 kD
95 kD
70 kD
55 kD
43 kD
33 kD
25 kD
17 kD
10 kD

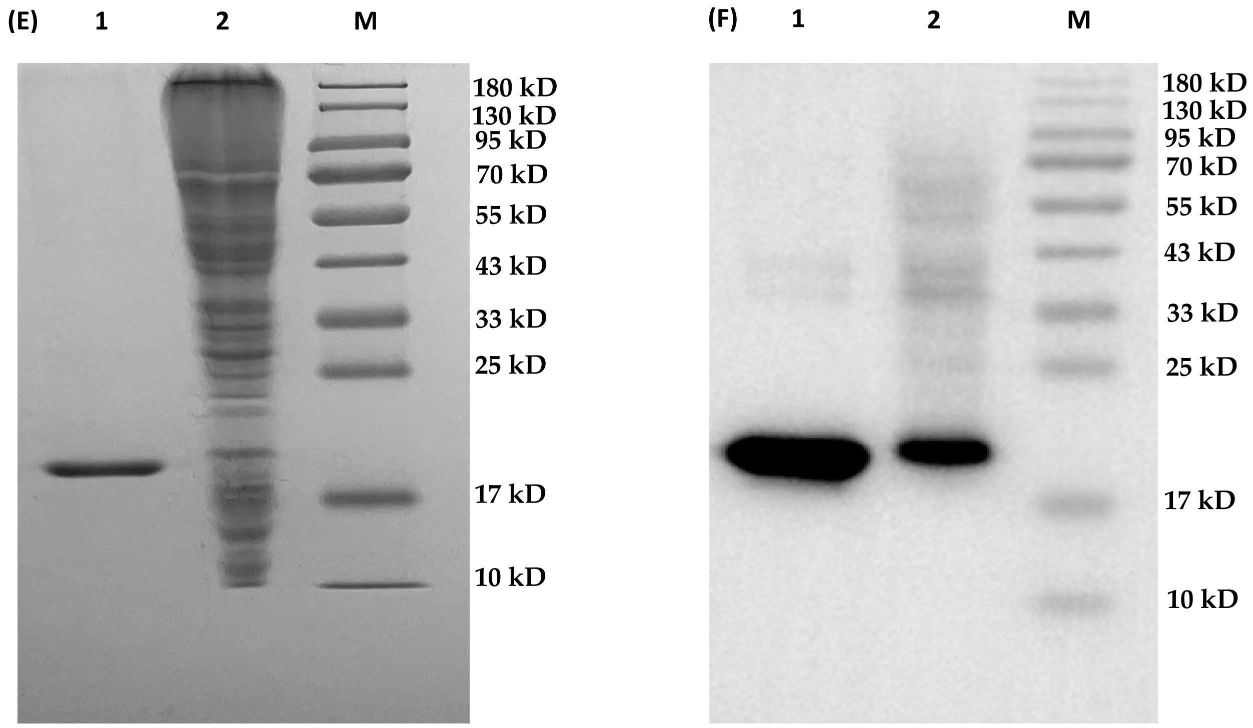


**Figure S3.** Identification of Nb-A12, Nb-B10 and Nb-C23 expression. Analysis of SDS-PAGE **(A)** and Western Blot **(B)** for Nb-A12; Analysis of SDS-PAGE **(C)** and Western Blot **(D)** for Nb-B10; Analysis of SDS-PAGE **(E)** and Western Blot **(F)** for Nb-C23; Ladder 1: Nanobody after purification on nickel column; Ladder 2: Total protein extract after induction; M: Marker.

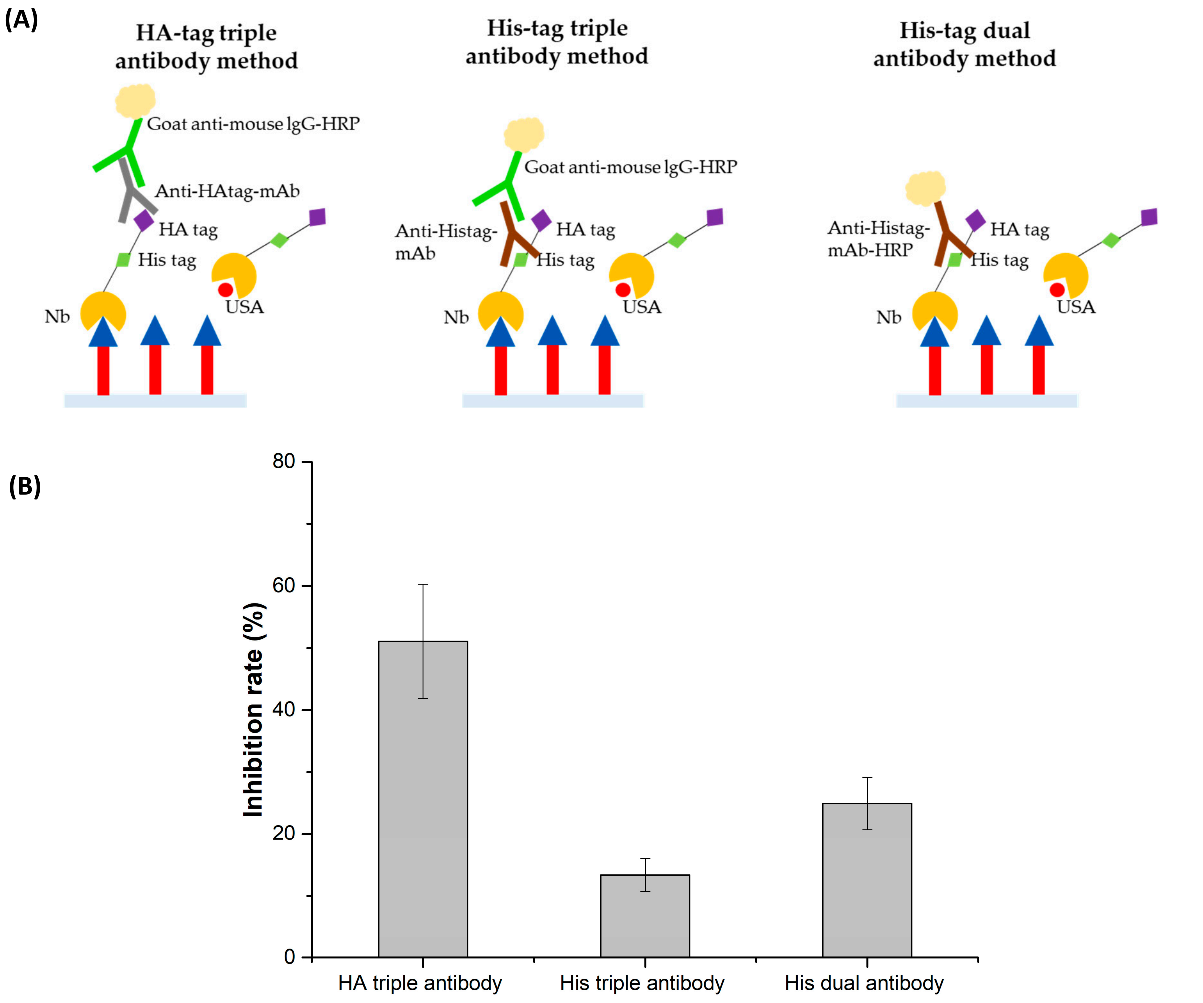


**Figure S4**. Schematic detections with different icELISAs and their inhibition rates. **(A)** Diagram of different detection forms; **(B)** Inhibition rates.

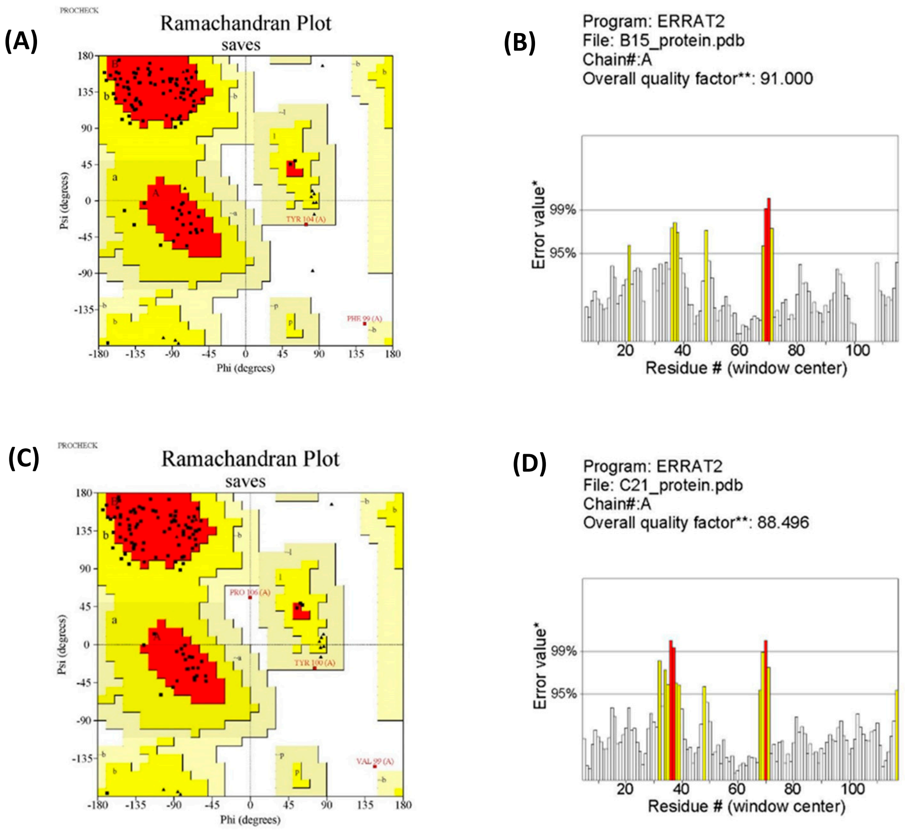


**Figure S5.** Quality evaluation of Nb-B15 and Nb-C21 models. (**A**) Ramachandran plot of the Nb-B15 model; (**B**) ERRAT of the Nb-B15 model; (**C**) Ramachandran plot of the Nb-C21 model; (**D**) ERRAT of the Nb-C21 model. The asterisks (**) in (B) and (D) are expressed as the percentage of the protein for which the calculated error value falls below the 95% rejection limit. Good high resolution structures generally produce values around 95% or higher. For lower resolutions (2.5 to 3 Å), the average overall quality factor is around 91%.

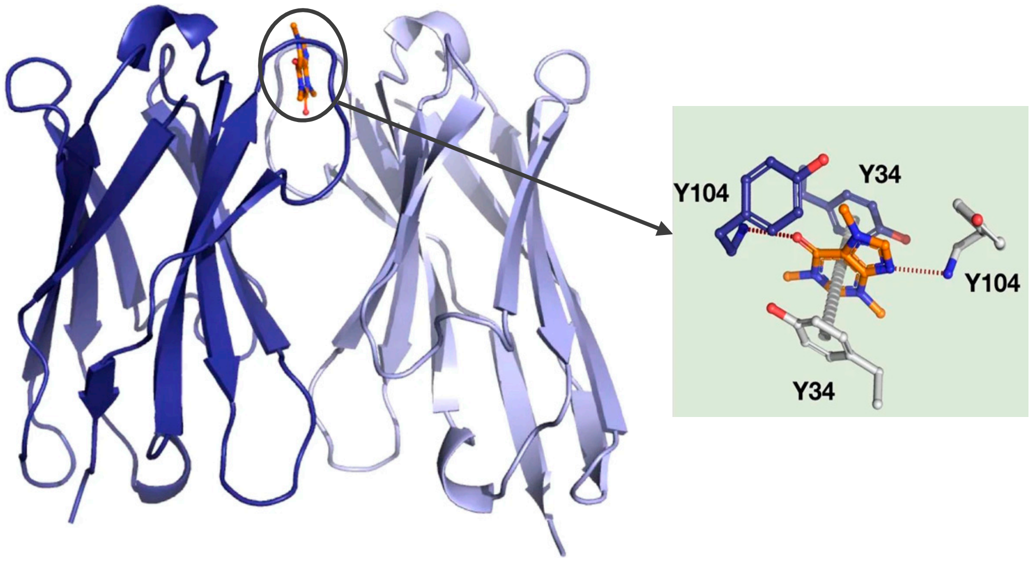


**Figure S6**. Complex of AcVHH dimer with caffeine. The x-ray structure of acVHH dimer is in cartoon style, and caffeine is in orange/blue/red stick style [47].

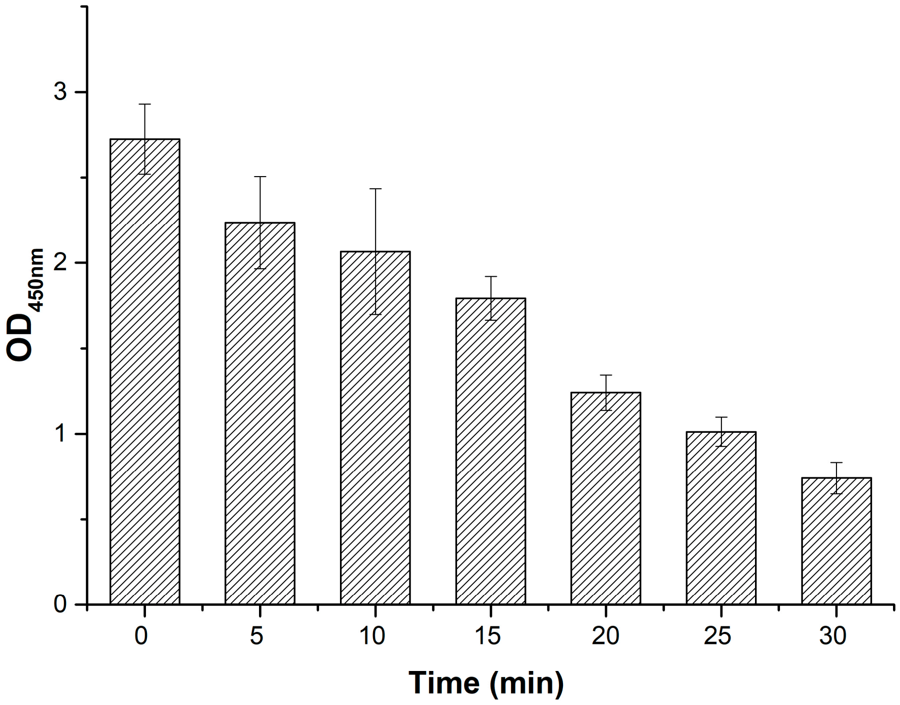


**Figure S7.** Thermo-stability of Nb-B15 treated at 50 °C in different time.

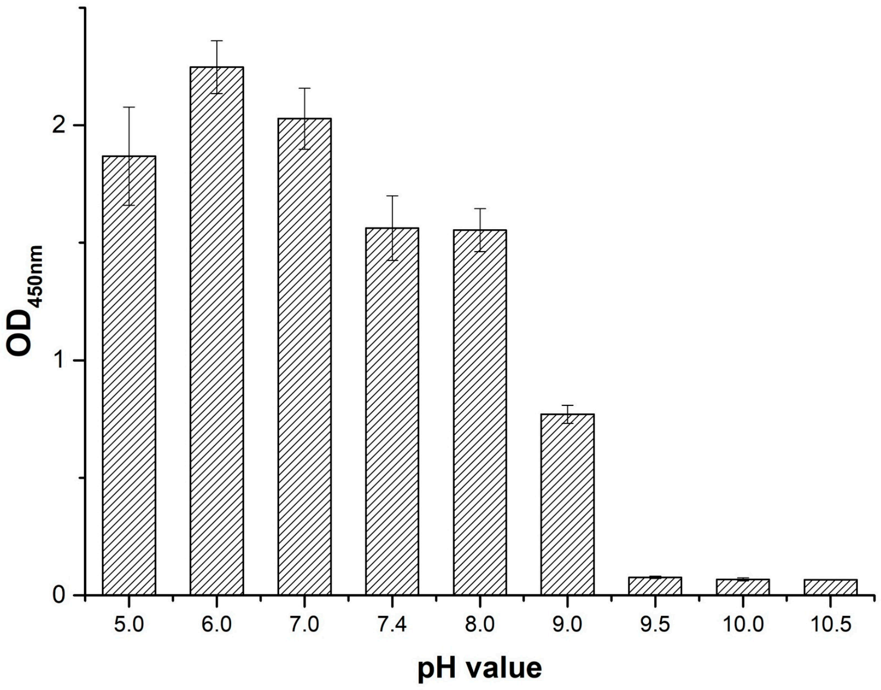


**Figure S8.** Effects of different pH values on the performance of Nb-B15-based ELISA.

**Table S1.** Titer and inhibition by ustilaginoidin A of alpaca serum after immunization.

| Booster Immunization | Titer of Serum | Inhibition Rate by Ustilaginoidin A at 1000 ng/mL (%) |
|---|---|---|
| Blank control | $<1.1\times10^2$ | 9.1 |
| 5th | $8\times10^3$ | 56 |
| 6th | $1.6\times10^4$ | 73 |
| 7th | $3.2\times10^4$ | 90 |

**Supplementary References**

47. Lesne, J.; Chang, H.; De, V.; Paloni, M.; Barthe, P.; Guichou, J.; Mayonove, P.; Barducci, A.; Labesse, G.; Bonnet, J.; Cohen, M. Structural basis for chemically-induced homodimerization of a single domain antibody. *Sci. Rep*. **2019**, *9*, 1840.
